# Data article on elemental and metabolomic-based alkaloidal composition in black pepper oleoresin using a positive ESI-mode LC-QToF and ICP-mass spectroscopy

**DOI:** 10.1016/j.dib.2018.06.034

**Published:** 2018-06-26

**Authors:** Olusegun Abayomi Olalere, Nour Hamid Abdurahman, Rosli bin Mohd Yunus, Oluwaseun Ruth Alara

**Affiliations:** a Centre of Excellence for Advanced Research in Fluid Flow, University Malaysia Pahang, Pahang, Malaysia; b Faculty of Chemical Engineering and Natural Resources, Universiti Malaysia Pahang, Lebuhraya Tun Razak, Gambang 26300, Malaysia

## Abstract

This paper contains data from the elemental and phytochemical profiling of black pepper oleoresin extracts using the LC–MS QToF and ICP-MS analysis. In recent years studies have shown the medicinal properties of extracts from these two cultivars of *Piper nigrum*. The medicinal properties are attributed to the presence of many secondary metabolites and mineral element in them. The phytochemical profiling was conducted using a Liquid Chromatography equipped with an electrospray time-of-flight mass spectrometer detectors. The mass spectrometer was equipped with an electrospray ionization sources operated in positive ion mode. The alkaloid compounds in the optimized black pepper extract were tentatively characterized in accordance with their ions׳ mass fragmentation.

**Specification Table**

**Table:** 

Subject area	Natural product research
More specific subject area	Metabolomics, medicinal chemistry
Type of data	Table, Figure
How data was acquired	a) Secondary metabolites dataset were acquired from the LC–MS-Quadrupled time of flight mass spectrometer online database (Vion Ion Mobility QTOF MS, Waters, USA). b) The mineral element composition was obtained from the output file of Inductively Coupled Plasma/Mass Spectrometer (ICP-MS 7500, Agilent, US)
Data source location	Centre of Excellence for Advanced Research in Fluid Flow, University Malaysia Pahang, Pahang, Malaysia
Data accessibility	Data is provided with this article (Supplementary file Table 1, Fig. 1 and Table 2)

**Value of the data**

● This data provides information about the alkaloid profiles in black pepper using metabolomics approach.
● The data provided the exact concentration of mineral element inside the black pepper.
● The information from the LC–MS/QToF and ICP-MS datasets provide a basis for future isolation of targeted compounds in black pepper.

## 1 Data

This dataset comprises of the secondary metabolites and mineral elements obtained from the Liquid Chromatography (LC–MS QToF) and Inductively Coupled Plasma Mass Spectrometry (ICP-MS) analysis of oleoresin extracted from black pepper. The output file from the database search of LC–MS/QToF is available in Supplementary Table 1. However the result from the mineral element analysis is available in Supplementary Table 2.

## 2 Material, method and sample preparation

### 2.1 Sample preparation

The microwave reflux extraction was carried out using a programmable microwave lab-stationed system (Ethos-ATC-FO 300, Milestone, North America). The extraction system is time, temperature and power-controlled. It is operated at 2.45 GHz with a maximum power output of 1000 W consisting of a cooling system, infrared optic fiber, microwave hole, temperature control and electromagnetic radiation. A precisely weighed 25 g of powdered black pepper was loaded into the microwave reactor and operated at optimum condition as reported by Olalere et al. [1]. Microwave extraction was conducted at 120 min of irradiation time, 350 W of microwave power, 0.105 mm of particle size and 1:12 of a feed-solvent ratio. After extraction, the reactor was unloaded from the microwave cavity and the sample filtered using vacuum pump (BUCHI V-100 model, Germany) and concentrated with rotary evaporator (BUCHI, R-200 model, Germany). The concentrated extracts were collected and stored in a dark vial bottles for subsequent physicochemical characterizations.

### 2.2 LC–MS/QToF analysis

The oleoresin stock solution was prepared for LC–MS analysis by dissolving the extract in methanol (HPLC, grade). The mixture was made up to a final concentration of 100 mg/mL. The extract was pre-treated and the concentration adjusted to 20 ppm before injection into the LC–MSQ- time of flight mass spectrometer. The mobile phase was prepared using the binary solvent manager with solvent A and B. The solvent A is made of 0.1% formic acid (Sigma Aldrich®, Germany) plus water (Milli-Q grade, v/v). However, solvent B was 0.1% formic acid in acetonitrile at a seal wash time and highest pressure limit of 5 min and 1800 psi, respectively [2]. The two solvents were then passed through the vacuum degasser. The gradient parameters are presented (Table 1).

**Table 1:** Gradient parameters in mobile phase preparation.

Time (min)	Flow rate (mL/min)	Composition A (%)	Composition B (%)
0.00	0.600	50	50
0.46	0.600	40	60
1.16	0.600	50	50
1.85	0.600	90	10
3.47	0.600	30	70

The bioactive compounds inside the black oleoresin extracts were identified using LC–MS-Quadrupled time of flight mass spectrometer (Vion Ion Mobility QTOF MS, Waters, USA). The parameters used in the operation of the LC–MS QTOF analysis are presented (Table 2).

**Table 2:** Operating parameters of Vion IMS QToF analyzer.

Operating parameters	Values
Source temperature	120 °C
Source type	ESI
Desolvation flow rate	800 L/h
Desolvation temperature	550 °C
Operation mode (Polarity)	Positive (–ve)
Analyzer Mode	Sensitivity
Cone gas	50 L/h
Scan time	0.200 s to 4.00 min
Lock correction interval	0.50 mm
MS mode	High definition
Collision energy interval	4.00–45.00 eV
Scanning range	100–1000 m/z
Column type	ACQUITY UPLCHSS T3
Dimension	2.1×100 mm, particle size 1.8 µm

### 2.3 ICP-MS trace-element analysis

The analysis of mineral elements (Na, Mg, K, Ca, Cr, Mn, Fe, Cu, Zn, As, Se, Cd, Pb) in black pepper oleoresin was carried out using an Inductively Coupled Plasma/Mass Spectrometer (ICP-MS 7500, Agilent, US) in accordance with the method used by Wati et al. [3]. A multi-element standard solution was used in the preparation of a calibration solution. A calibration curve was constructed from five concentrations (0–50 ppm) of the standard solution using 2% of nitric acid (HNO_3_) as blank. The liquid sample was introduced into the ICP-MS nebulizer and spray chamber. The sample was dried, vaporized, atomized and ionized inside the plasma chamber consisting of different heating zones. The elemental composition of the sample was obtained from the transformation of the liquid samples into excited atoms and positively charged ions.
