# Supplementary material for: Data article on elemental and metabolomic-based alkaloidal composition in black pepper oleoresin using a positive ESI-mode LC-QToF and ICP-mass spectroscopy
Source: Data Brief. 2018 Jun 26;19:1627–30. doi: 10.1016/j.dib.2018.06.034 (PMC6141158; doi:10.1016/j.dib.2018.06.034)
Supplement: Supplementary file 2 — Supplementary material [file mmc2.docx]

**
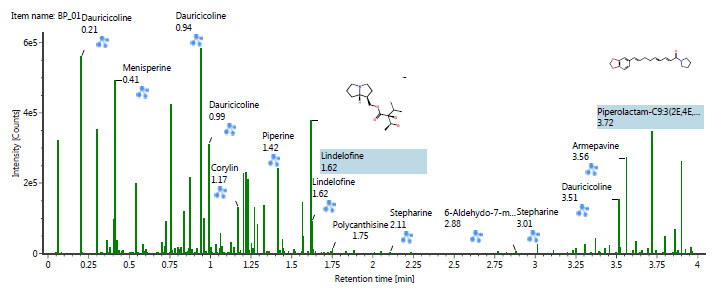
**

**Fig.1** LC-QToF analysis of optimized oleoresin extract of black pepper by microwave reflux extraction method

**Table 1**

Identified alkaloid in the black pepper extract

| S/N | Component name | | Formula | | Status | Observed m/z | | Observed RT  (min) | | Adducts | | Total Fragments Found | |
| --- | --- | --- | --- | --- | --- | --- | --- | --- | --- | --- | --- | --- | --- |
| 1 | δ-Humulene | | C_15_H_22_ | | - | - | | - | | - | | - | |
| 2 | Piperolein B | | C_21_H_29_NO_3_ | | Identified | 366.2055 | | 0.05 | | +Na | | 0 | |
| 3 | Piperolein B | | C_21_H_29_NO_3_ | | Identified | 344.2220 | | 0.06 | | +H,  +Na | | 20 | |
| 4 | Thebaine | | C_19_H_21_NO_3_ | | Identified | 312.1593 | | 0.08 | | +H | | 14 | |
| 5 | Dauricicoline | | C_36_H_40_N_2_O _6_ | | Identified | 597.2959 | | 0.21 | | +H,  +Na | | 40 | |
| 6 | Delbruline | | C_26_H_41_NO_7_ | | Identified | 502.2780 | | 0.21 | | +Na, +K | | 12 | |
| 7 | Dauricicoline | | C_36_H_40_N_2_O _6_ | | Identified | 635.2521 | | 0.30 | | +K | | 4 | |
| 8 | Picrasidine R | | C_30_H_26_N_4_O _6_ | | Identified | 561.1739 | | 0.33 | | +Na | | 1 | |
| 9 | Bruceine A | | C_26_H_34_O_11_ | | Identified | 523.2161 | | 0.33 | | +H, +K | | 21 | |
| 10 | Isotetrandrine | | C_38_H_42_N_2_O _6_ | | Identified | 661.2679 | | 0.36 | | +K, +H | | 11 | |
| 11 | Trigonelline | | C_7_H_7_NO_2_ | | Identified | 138.0551 | | 0.37 | | +H | | 0 | |
| 12 | Neojiangyouaconitine | | C_33_H_47_NO_9_ | | Identified | 624.3153 | | 0.38 | | +Na | | 4 | |
| 13 | Longicamphenylone | | C_14_H_22_O | | Identified | 229.1547 | | 0.38 | | +Na | | 1 | |
| 14 | Dihydrogentianine | | C_10_H_11_NO_2_ | | Identified | 178.0861 | | 0.38 | | +H | | 8 | |
| 15 | Chamigrenal | | C_15_H_22_O | | Identified | 241.1550 | | 0.39 | | +Na | | 0 | |
| 16 | Armepavine | | C_19_H_23_NO_3_ | | Identified | 314.1752 | | 0.40 | | +H | | 46 | |
| 17 | Menisperine | | C_21_H_25_NO_4_ | | Identified | 356.1857 | | 0.41 | | +H | | 35 | |
| 18 | Codeine | | C_18_H_21_NO_3_ | | Identified | 300.1604 | | 0.42 | | +H | | 49 | |
| 19 | Sinomenine | | C_19_H_23_NO_4_ | | Identified | 330.1703 | | 0.42 | | +H,  +Na | | 98 | |
| 20 | 6-Hydroxy-2-[2-(4'- methoxyphenyl)ethyl] chromone | | C_18_H_16_O_4_ | | Identified | 297.1123 | | 0.42 | | +H | | 15 | |
| 21 | Nigakilactone E | | C_24_H_34_O_8_ | | Identified | 473.2134 | | 0.44 | | +Na | | 89 | |
| 22 | Cimicifugic acid B | | C_21_H_20_O_11_ | | Identified | 449.1070 | | 0.44 | | +H | | 31 | |
| 23 | Fawcettiine | | C_18_H_29_NO_3_ | | Identified | 308.2226 | | 0.45 | | +H | | 22 | |
| 24 | Resokaempferol | | C_15_H_10_O_5_ | | Identified | 271.0600 | | 0.46 | | +H | | 3 | |
| 25 | Resokaempferol | | C_15_H_10_O_5_ | | Identified | 271.0599 | | 0.47 | | +H | | 2 | |
| 26 | Delbruline | | C_26_H_41_NO_7_ | | Identified | 502.2762 | | 0.51 | | +Na | | 62 | |
| 27 | Delbruline | | C_26_H_41_NO_7_ | | Identified | 502.2784 | | 0.52 | | +Na | | 46 | |
| 28 | Morusinol | | C_25_H_26_O_7_ | | Identified | 461.1569 | | 0.52 | | +Na | | 38 | |
| 29 | N-cis-Feruloyl typamine | | C_18_H_19_NO_4_ | | Identified | 314.1388 | | 0.54 | | +H,  +Na | | 18 | |
| 30 | Epiberberine | | C_20_H_17_NO_4_ | | Identified | 358.1060 | | 0.54 | | +Na | | 6 | |
| 31 | Erythrocentaurin | | C_10_H_8_O_3_ | | Identified | 177.0545 | | 0.54 | | +H | | 8 | |
| 32 | Erythrocentaurin | | C_10_H_8_O_3_ | | Identified | 177.0545 | | 0.55 | | +H | | 3 | |
| 33 | Ingenol-3- hexadecanoate | | C_36_H_58_O_6_ | | Identified | 609.4121 | | 0.56 | | +Na | | 71 | |
| 34 | Methyl kushenol C | | C_26_H_28_O_7_ | | Identified | 475.1743 | | 0.56 | | +Na | | 31 | |
| 35 | Flavenochromane B | | C_25_H_26_O_6_ | | Identified | 445.1629 | | 0.58 | | +Na | | 24 | |
| 36 | Turpinionosides B | | C_19_H_34_O_8_ | | Identified | 413.2146 | | 0.58 | | +Na | | 57 | |
| 37 | Bruceine A | | C_26_H_34_O_11_ | | Identified | 561.1730 | | 0.59 | | +K, +Na | | 97 | |
| 38 | 7,8-  Dimethoxymyrtopsine | | C_17_H_21_NO_6_ | | Identified | 358.1264 | | 0.59 | | +Na, +K | | 15 | |
| 39 | 1β,3β,6α-Trihydroxy-4α (15)-dihydrocostic acid methyl ester-1-O-β-D- glucopyranoside | | C_22_H_36_O_10_ | | Identified | 499.1951 | | 0.60 | | +K, +Na | | 75 | |
| 40 | Bruceine I | | C_22_H_28_O_9_ | | Identified | 475.1380 | | 0.61 | | +K, +Na | | 81 | |
| 41 | Qingdainone | | C_23_H_13_N_3_O_2_ | | Identified | 402.0643 | | 0.61 | | +K | | 5 | |
| 42 | 4,7-  Didehydroneophysalin B | | C_28_H_28_O_9_ | | Identified | 531.1632 | | 0.61 | | +Na | | 56 | |
| 43 | Galangin (Norizalpinin) | | C_15_H_10_O_5_ | | Identified | 271.0609 | | 0.67 | | +H | | 4 | |
| 44 | Corylin | | C_20_H_16_O_4_ | | Identified | 321.1120 | | 0.67 | | +H | | 13 | |
| 45 | Qingdainone | | C_23_H_13_N_3_O_2_ | | Identified | 402.0649 | | 0.68 | | +K, +Na | | 9 | |
| 46 | Nigakilactone E | | C_24_H_34_O_8_ | | Identified | 489.1891 | | 0.70 | | +K, +Na | | 68 | |
| 47 | Methoxychelidonine | | C_21_H_21_NO_6_ | | Identified | 384.1422 | | 0.70 | | +H | | 39 | |
| 48 | Erythrocentaurin | | C_10_H_8_O_3_ | | Identified | 177.0549 | | 0.70 | | +H | | 2 | |
| 49 | N-trans-Feruloyl piperidine | | C_15_H_19_NO_3_ | | Identified | 262.1440 | | 0.70 | | +H | | 3 | |
| 50 | Codonopsine | | C_14_H_21_NO_4_ | | Identified | 268.1525 | | 0.70 | | +H | | 45 | |
| 51 | Anisodamine | | C_17_H_23_NO_4_ | | Identified | 306.1691 | | 0.71 | | +H | | 67 | |
| 52 | Hordenine-Ο-α-L- rhamnopyranoside | | C_16_H_25_NO_5_ | | Identified | 334.1630 | | 0.72 | | +Na | | 47 | |
| 53 | d-Isoboldine | | C_19_H_21_NO_4_ | | Identified | 328.1545 | | 0.73 | | +H | | 28 | |
| 54 | Menisperine | | C_21_H_25_NO_4_ | | Identified | 356.1863 | | 0.73 | | +H | | 33 | |
| 55 | N-cis-Feruloyl typamine | | C_18_H_19_NO_4_ | | Identified | 314.1394 | | 0.75 | | +H | | 17 | |
| 56 | Isotetrandrine | | C_38_H_42_N_2_O _6_ | | Identified | 623.3114 | | 0.76 | | +H, +K,  +Na | | 96 | |
| 57 | Piperolein B | | C_21_H_29_NO_3_ | | Identified | 344.2224 | | 0.76 | | +H | | 24 | |
| 58 | Anisodamine | | C_17_H_23_NO_4_ | | Identified | 306.1683 | | 0.78 | | +H | | 22 | |
| 59 | Cryptopine | | C_21_H_23_NO_5_ | | Identified | 370.1630 | | 0.78 | | +H,  +Na | | 36 | |
| 60 | (E)-1,3-Dihydro-3-[(4-  hydroxy-3,5- dimethoxyphenyl) methylene]-2H-indol-2- one | | C_17_H_15_NO_4_ | | Identified | 298.1066 | | 0.79 | | +H | | 2 | |
| 61 | Norisocorydine | | C_19_H_21_NO_4_ | | Identified | 328.1545 | | 0.80 | | +H | | 20 | |
| 62 | | Aristolactam A | C_16_H_11_NO3 | Identified | | | 266.0812 | | 0.81 | | +H | | 2 |
| 63 | | Hyoscyamine | C_17_H_23_NO3 | Identified | | | 290.1750 | | 0.82 | | +H | | 20 |
| 64 | | Hordatine B | C_29_H_40_N_8_O _5_ | Identified | | | 581.3194 | | 0.84 | | +H | | 75 |
| 65 | | Hordenine-Ο-α-L- rhamnopyranoside | C_16_H_25_NO_5_ | Identified | | | 334.1624 | | 0.84 | | +Na, +K | | 53 |
| 66 | | Feruperine | C_17_H_21_NO3 | Identified | | | 288.1596 | | 0.84 | | +H | | 15 |
| 67 | | Supinin | C_15_H_25_NO_4_ | Identified | | | 306.1674 | | 0.85 | | +Na | | 27 |
| 68 | | Sophoflavescenol | C_22_H_24_O_6_ | Identified | | | 385.1653 | | 0.86 | | +H | | 39 |
| 69 | | Jesaconitine | C_35_H_49_NO_12_ | Identified | | | 714.2906 | | 0.86 | | +K | | 98 |
| 70 | | Isocorynoxeine | C_22_H_26_N_2_O_4_ | Identified | | | 405.1787 | | 0.87 | | +Na, +K | | 54 |
| 71 | | Dihydropalmatine | C_21_H_23_NO4 | Identified | | | 354.1691 | | 0.87 | | +H | | 13 |
| 72 | | Isotetrandrine | C_38_H_42_N_2_O_6_ | Identified | | | 623.3105 | | 0.87 | | +H | | 50 |
| 73 | | Songorine | C_22_H_31_NO3 | Identified | | | 358.2376 | | 0.88 | | +H | | 32 |
| 74 | | Leonticine | C_20_H_25_NO3 | Identified | | | 328.1906 | | 0.89 | | +H | | 29 |
| 75 | | N-Oxy- tuberostemonine | C_22_H_33_NO_5_ | Identified | | | 414.2251 | | 0.91 | | +Na | | 62 |
| 76 | | Qingdainone | C_23_H_13_N_3_O_2_ | Identified | | | 402.0634 | | 0.91 | | +K | | 3 |
| 77 | | Piperlactam S | C_17_H_13_NO_4_ | Identified | | | 296.0916 | | 0.92 | | +H | | 8 |
| 78 | | Magnocurarine | C_19_H_24_NO_3_ | Identified | | | 353.1385 | | 0.92 | | +K | | 26 |
| 79 | | Dauricicoline | C_36_H_40_N_2_O_6_ | Identified | | | 635.2519 | | 0.94 | | +K, +H,  +Na | | 101 |
| 80 | | Meteloidine | C_13_H_21_NO_4_ | Identified | | | 278.1364 | | 0.96 | | +Na | | 7 |
| 81 | | Meteloidine | C_13_H_21_NO_4_ | Identified | | | 278.1364 | | 0.96 | | +Na | | 15 |
| 82 | | Piperyline | C_16_H_17_NO_3_ | Identified | | | 272.1283 | | 0.97 | | +H | | 9 |
| 83 | | Piperolactam-C5:1(2E) | C_16_H_19_NO_3_ | Identified | | | 274.1431 | | 0.97 | | +H | | 4 |
| 84 | | Sinomenine | C_19_H_23_NO_4_ | Identified | | | 330.1704 | | 0.99 | | +H | | 39 |
| 85 | | Dauricicoline | C_36_H_40_N_2_O_6_ | Identified | | | 619.2780 | | 0.99 | | +Na | | 33 |
| 86 | | Platydesmine | C_15_H_17_NO_3_ | Identified | | | 260.1283 | | 1.00 | | +H | | 2 |
| 87 | | 7,8-  Dimethoxyplatydesmine | C_17_H_21_NO_5_ | Identified | | | 320.1474 | | 1.01 | | +H,  +Na | | 25 |
| 88 | | Isodidehydrotubero- stemonine | C_22_H_29_NO_4_ | Identified | | | 372.2170 | | 1.02 | | +H | | 42 |
| 89 | | α-Allocryptopine | C_21_H_23_NO_5_ | Identified | | | 370.1650 | | 1.03 | | +H | | 29 |

| 90 | Piperlongumine | C_17_H_19_NO_5_ | Identified | 340.1156 | 1.04 | +Na,  +H | 41 |
| --- | --- | --- | --- | --- | --- | --- | --- |
| 91 | Cryptopine | C_21_H_23_NO_5_ | Identified | 370.1652 | 1.04 | +H | 35 |
| 92 | Meteloidine | C_13_H_21_NO_4_ | Identified | 278.1364 | 1.06 | +Na | 8 |
| 93 | Meteloidine | C_13_H_21_NO_4_ | Identified | 278.1364 | 1.07 | +Na | 16 |
| 94 | Yuanhunine | C_21_H_25_NO_4_ | Identified | 356.1860 | 1.07 | +H | 15 |
| 95 | Morusinol | C_25_H_26_O_7_ | Identified | 439.1736 | 1.07 | +H | 39 |
| 96 | Piperolein B | C_21_H_29_NO_3_ | Identified | 344.2226 | 1.07 | +H | 12 |
| 97 | Stemospironine | C_19_H_29_NO_5_ | Identified | 374.1935 | 1.08 | +Na | 31 |
| 98 | Piperolactam-C5:1(2E) | C_16_H_19_NO_3_ | Identified | 274.1437 | 1.11 | +H | 5 |
| 99 | Meteloidine | C_13_H_21_NO_4_ | Identified | 278.1369 | 1.12 | +Na | 13 |
| 100 | Meteloidine | C_13_H_21_NO_4_ | Identified | 278.1368 | 1.12 | +Na | 19 |
| 101 | Pipernonaline | C_21_H_27_NO_3_ | Identified | 342.2064 | 1.14 | +H | 14 |
| 102 | Sophoranodichromane A | C_25_H_28_O_6_ | Identified | 447.1779 | 1.17 | +Na | 10 |
| 103 | Corylin | C_20_H_16_O_4_ | Identified | 321.1124 | 1.17 | +H | 19 |
| 104 | 13,13a-Didehydro-9, 10- dimethoxy-2,3- (ethylenedioxy)-berbine | C_20_H_19_NO_4_ | Identified | 338.1391 | 1.17 | +H | 15 |
| 105 | Saurolactam | C_17_H_13_NO_3_ | Identified | 280.0970 | 1.18 | +H | 4 |
| 106 | Dauricicoline | C_36_H_40_N_2_O _6_ | Identified | 597.2960 | 1.20 | +H | 60 |
| 107 | Hordenine-Ο-α-L- rhamnopyranoside | C_16_H_25_NO_5_ | Identified | 334.1627 | 1.21 | +Na | 48 |
| 108 | Hyoscyamine | C_17_H_23_NO_3_ | Identified | 312.1590 | 1.22 | +Na | 29 |
| 109 | Menisperine | C_21_H_25_NO_4_ | Identified | 356.1857 | 1.22 | +H | 31 |
| 110 | Oliveridine | C_19_H_19_NO_4_ | Identified | 326.1382 | 1.22 | +H | 35 |
| 111 | Dauricicoline | C_36_H_40_N_2_O _6_ | Identified | 597.2960 | 1.22 | +H, +K,  +Na | 67 |
| 112 | Isotetrandrine | C_38_H_42_N_2_O _6_ | Identified | 623.3114 | 1.23 | +H, +K,  +Na | 89 |
| 113 | Meteloidine | C_13_H_21_NO_4_ | Identified | 278.1362 | 1.23 | +Na | 21 |
| 114 | Isosinomenine | C_19_H_23_NO_4_ | Identified | 330.1692 | 1.23 | +H | 41 |
| 115 | Meteloidine | C_13_H_21_NO_4_ | Identified | 278.1363 | 1.24 | +Na | 15 |
| 116 | Pipernonaline | C_21_H_27_NO_3_ | Identified | 342.2063 | 1.24 | +H | 20 |
| 117 | Rugosinone | C_19_H_15_NO_6_ | Identified | 354.0972 | 1.25 | +H,  +Na | 12 |
| 118 | N-Oxy- tuberostemonine | C_22_H_33_NO_5_ | Identified | 414.2252 | 1.25 | +Na, +K | 47 |

| 119 | Hordatine B | C_29_H_40_N_8_O _5_ | Identified | 581.31 | 1.26 | +H,  +Na | 167 |
| --- | --- | --- | --- | --- | --- | --- | --- |
| 120 | Piperanine | C_17_H_21_NO_3_ | Identified | 288.15 | 1.27 | +H,  +Na | 12 |
| 121 | Scopolamine | C_17_H_21_NO_4_ | Identified | 304.1525 | 1.29 | +H | 2 |
| 122 | Scopolamine | C_17_H_21_NO_4_ | Identified | 304.1525 | 1.29 | +H | 24 |
| 123 | Scopolamine | C_17_H_21_NO_4_ | Identified | 304.1525 | 1.29 | +H | 19 |
| 124 | Morphine | C_17_H_19_NO_3_ | Identified | 286.1442 | 1.33 | +H | 8 |
| 125 | Stemospironine | C_19_H_29_NO_5_ | Identified | 352.2103 | 1.35 | +H | 11 |
| 126 | 3-Dimethylallyl-4- methoxy-2-quinolone | C_15_H_17_NO_2_ | Identified | 244.13 | 1.36 | +H | 2 |
| 127 | Anisodamine | C_17_H_23_NO_4_ | Identified | 306.1681 | 1.36 | +H | 18 |
| 128 | Isotetrandrine | C_38_H_42_N_2_O _6_ | Identified | 623.31 | 1.41 | +H, +K | 32 |
| 129 | Piperine | C_17_H_19_NO_3_ | Identified | 286.1442 | 1.42 | +H | 7 |
| 130 | Stepharine | C_18_H_19_NO_3_ | Identified | 298.14 | 1.43 | +H,  +Na | 12 |
| 131 | Scopolamine | C_17_H_21_NO_4_ | Identified | 304.1525 | 1.44 | +H | 31 |
| 132 | 5-O-Methylvisamminol | C_16_H_18_O_5_ | Identified | 313.1036 | 1.45 | +Na | 4 |
| 133 | 5-O-Methylvisamminol | C_16_H_18_O_5_ | Identified | 313.1037 | 1.45 | +Na | 11 |
| 134 | Hirsutine | C_22_H_28_N_2_O_3_ | Identified | 391.20 | 1.48 | +Na | 22 |
| 135 | Anthranoyllycoctonine | C_32_H_46_N_2_O_8_ | Identified | 625.28 | 1.51 | +K | 47 |
| 136 | 2′,4′-Dihydroxy-4,6′- dimethoxy- dihydrochalcone | C_17_H_18_O_5_ | Identified | 325.10 | 1.52 | +Na | 2 |
| 137 | 5-O-Methylvisamminol | C_16_H_18_O_5_ | Identified | 313.1035 | 1.56 | +Na | 7 |
| 138 | Preskimmianine | C_17_H_21_NO_4_ | Identified | 304.1524 | 1.57 | +H | 10 |
| 139 | Scopolamine | C_17_H_21_NO_4_ | Identified | 304.1524 | 1.57 | +H | 18 |
| 140 | Sinapine | C_16_H_23_NO_5_ | Identified | 332.1472 | 1.60 | +Na | 23 |
| 141 | Armepavine | C_19_H_23_NO_3_ | Identified | 314.1752 | 1.62 | +H | 14 |
| 142 | Piperolactam-C7:1(6E) | C1_8_H_23_NO_3_ | Identified | 302.1757 | 1.62 | +H | 12 |
| 143 | Lindelofine | C_15_H_27_NO_4_ | Identified | 308.1836 | 1.62 | +Na, +K | 16 |
| 144 | Piperolactam-C7:1(6E) | C_18_H_23_NO_3_ | Identified | 302.18 | 1.62 | +H,  +Na | 23 |
| 145 | Lindelofine | C_15_H_27_NO_4_ | Identified | 308.1836 | 1.62 | +Na | 4 |
| 146 | Thebaine | C_19_H_21_NO_3_ | Identified | 334.1404 | 1.63 | +Na | 11 |

| 147 | Thebaine | C_19_H_21_NO_3_ | Identified | | | 334.1419 | 1.63 | +Na,  +H | | 34 |
| --- | --- | --- | --- | --- | --- | --- | --- | --- | --- | --- |
| 148 | Nobilonine | C_17_H_27_NO_3_ | Identified | | | 294.2045 | 1.65 | +H | | 24 |
| 149 | Codeine | C_18_H_21_NO_3_ | Identified | | | 300.1598 | 1.68 | +H | | 14 |
| 150 | Piperyline | C_16_H_17_NO_3_ | Identified | | | 272.1287 | 1.69 | +H | | 3 |
| 151 | Piperyline | C_16_H_17_NO_3_ | Identified | | | 272.1286 | 1.69 | +H | | 6 |
| 152 | Fuziline (15-α- Hydroxyneoline) | C_24_H_39_NO_7_ | Identified | | | 476.2616 | 1.70 | +Na | | 26 |
| 153 | Pipernonaline | C_21_H_27_NO_3_ | Identified | | | 342.2064 | 1.70 | +H | | 10 |
| 154 | 5-O-Methylvisamminol | C_16_H_18_O_5_ | Identified | | | 313.1034 | 1.72 | +Na | | 2 |
| 155 | Pronuciferine | C_19_H_21_NO_3_ | Identified | | | 312.1604 | 1.73 | +H | | 5 |
| 156 | Hordenine-Ο-α-L- rhamnopyranoside | C_16_H_25_NO_5_ | Identified | | | 334.1629 | 1.74 | +Na, +K | | 12 |
| 157 | Hordenine-Ο-α-L- rhamnopyranoside | C_16_H_25_NO_5_ | Identified | | | 334.1628 | 1.74 | +Na | | 3 |
| 158 | Stepholidine | C_19_H_21_NO_4_ | Identified | | | 328.1544 | 1.74 | +H,  +Na | | 11 |
| 159 | Neoline | C_24_H_39_NO_6_ | Identified | | | 460.2681 | 1.74 | +Na | | 22 |
| 160 | Polycanthisine | C_13_H_21_NO | Identified | | | 208.1699 | 1.75 | +H | | 0 |
| 161 | Sanggenon H | C_20_H_18_O_6_ | Identified | | | 377.0995 | 1.83 | +Na | | 15 |
| 162 | N-Oxy- tuberostemonine | C_22_H_33_NO_5_ | Identified | | | 392.2415 | 1.88 | +H | | 25 |
| 163 | 12-α-Hydroxyevodol | C_26_H_28_O_10_ | Identified | | | 501.1754 | 2.01 | +H | | 2 |
| 164 | Apohyoscine | C_17_H_19_NO_3_ | Identified | | | 308.1260 | 2.05 | +Na | | 2 |
| 165 | Stepharine | C_18_H_19_NO_3_ | Identified | | | 298.1439 | 2.11 | +H | | 1 |
| 166 | 8-C-Prenylkaempferol | C_20_H_18_O_6_ | Identified | | | 377.0997 | 2.24 | +Na | | 0 |
| 167 | Menisperine | C_21_H_25_NO_4_ | Identified | | | 356.1858 | 2.77 | +H | | 0 |
| 168 | Camphenilone | C_9_H_14_O | Identified | | | 177.0676 | 2.82 | +K | | 0 |
| 169 | 6-Aldehydo-7-methoxy- isoophiopogonone B | C_20_H_18_O_6_ | Identified | | | 377.0998 | 2.88 | +Na | | 1 |
| 170 | Stepharine | C_18_H_19_NO_3_ | Identified | | | 298.1440 | 3.01 | +H | | 1 |
| 171 | 12-α-Hydroxyevodol | C_26_H_28_O_1_0 | Identified | | | 501.1756 | 3.11 | +H | | 0 |
| 172 | Supinin | C_15_H_25_NO_4_ | Identified | | | 306.1678 | 3.20 | +Na | | 1 |
| 173 | Dicentrine | C_20_H_21_NO_4_ | Identified | | | 340.1547 | 3.23 | +H,  +Na | | 6 |
| 174 | Isodidehydrotubero- stemonine | C_22_H_29_NO_4_ | Identified | | | 372.2170 | 3.26 | +H,  +Na | | 8 |
| 175 | Morphine | C_17_H_19_NO_3_ | Identified | | | 286.1440 | 3.31 | +H | | 4 |
| 176 | 6,7-Dehydroartemisinic acid | C_15_H_20_O_2_ | | Identified | 255.1374 | | 3.34 | | +Na | 7 |
| 177 | Piperolein B | C_21_H_29_NO_3_ | | _Id_entified | 344.2220 | | 3.37 | | +H,  +Na | 23 |
| 178 | Xantholide A | C_15_H_18_O_2_ | | Identified | 231.1374 | | 3.37 | | +H | 9 |
| 179 | Supinin | C_15_H_25_NO_4_ | | Identified | 306.1677 | | 3.39 | | +Na, +K | 8 |
| 180 | Piperyline | C_16_H_17_NO_3_ | | Identified | 272.1285 | | 3.40 | | +H | 8 |
| 181 | Menisperine | C_21_H_25_NO_4_ | | Identified | 356.1856 | | 3.43 | | +H | 8 |
| 182 | Morphine | C_17_H_19_NO_3_ | | Identified | 286.1440 | | 3.46 | | +H | 5 |
| 183 | Chloranthalactone A | C_15_H_16_O_2_ | | Identified | 229.1223 | | 3.46 | | +H | 8 |
| 184 | Leonticine | C_20_H_25_NO_3_ | | Identified | 328.1913 | | 3.46 | | +H | 8 |
| 185 | Dihydropalmatine | C_21_H_23_NO_4_ | | Identified | 354.1702 | | 3.49 | | +H | 11 |
| 186 | d-Stylopine | C_19_H_17_NO_4_ | | Identified | 324.1212 | | 3.50 | | +H | 7 |
| 187 | Dauricicoline | C_36_H_40_N_2_O _6_ | | Identified | 597.2958 | | 3.51 | | +H | 61 |
| 188 | Bulbocapnine | C_19_H_19_NO_4_ | | Identified | 326.1387 | | 3.52 | | +H | 13 |
| 189 | 4-Hydroxy-3- butylphthalide | C_12_H_14_O_3_ | | Identified | 229.0855 | | 3.56 | | +Na | 12 |
| 190 | Armepavine | C_19_H_23_NO_3_ | | Identified | 314.1750 | | 3.56 | | +H,  +Na | 17 |
| 191 | Thebaine | C_19_H_21_NO_3_ | | Identified | 312.1594 | | 3.60 | | +H | 39 |
| 192 | Pronuciferine | C_19_H_21_NO_3_ | | Identified | 312.1594 | | 3.62 | | +H | 4 |
| 193 | Melazolide A | C_11_H_16_O_4_ | | Identified | 235.0942 | | 3.64 | | +Na | 6 |
| 194 | (3R,4S)-4',7-  Dimethoxy-3'- deoxysappanol | C_18_H_20_O_5_ | | Identified | 339.1193 | | 3.66 | | +Na | 2 |
| 195 | Armepavine | C_19_H_23_NO_3_ | | Identified | 314.1751 | | 3.69 | | +H | 13 |
| 196 | Hypoglaucin H | C_39_H_60_O_15_ | | Identified | 807.3567 | | 3.71 | | +K | 116 |
| 197 | Piperolactam-C9:3  (2E,4E,8E) | C_20_H_23_NO_3_ | | Identified | 326.1750 | | 3.72 | | +H,  +Na | 27 |
| 198 | 1-[(2E,4E)-2,4-Decadie-  noyl]pyrrolidine | C_14_H_23_NO | | Identified | 222.1852 | | 3.74 | | +H | 0 |
| 199 | (3R,4S)-4',7-  Dimethoxy-3'- deoxysappanol | C_18_H_20_O_5_ | | Identified | 339.1196 | | 3.79 | | +Na | 3 |
| 200 | Thebaine | C_19_H_21_NO_3_ | | Identified | 312.1594 | | 3.80 | | +H,  +Na | 20 |

| . | Component name | Formula | Identification status | Observed m/z | Observed RT  (min) | Adducts | Total Fragments Found |
| --- | --- | --- | --- | --- | --- | --- | --- |
| 201 | (2E,4E,8E)-9-(Benzo [d]  [1,3]dioxol-5-yl)-N- isobutylnona-2,4,8- trienamide | C_20_H_25_NO_3_ | Identified | 350.1710 | 3.85 | +Na | 8 |
| 202 | Isotetrandrine | C_38_H_42_N_2_O _6_ | Identified | 645.2940 | 3.86 | +Na | 12 |
| 203 | Carmichaeline | C_22_H_35_NO_4_ | Identified | 400.2465 | 3.86 | +Na,  +H, +K | 25 |
| 204 | Fuziline (15-α- Hydroxyneoline) | C_24_H_39_NO_7_ | Identified | 476.2637 | 3.87 | +Na | 19 |
| 205 | Talatisamine | C_24_H_39_NO_5_ | Identified | 444.2723 | 3.88 | +Na, +K | 32 |
| 206 | Dehydrolindestrenolide | C_15_H_16_O_2_ | Identified | 229.1220 | 3.90 | +H | 10 |
| 207 | (2E,4E,8E)-9-(Benzo [d]  [1,3]dioxol-5-yl)-N- isobutylnona-2,4,8- trienamide | C_20_H_25_NO_3_ | Identified | 328.1908 | 3.90 | +H,  +Na | 27 |
| 208 | Dehydrolindestrenolide | C_15_H_16_O_2_ | Identified | 229.1220 | 3.90 | +H | 19 |
| 209 | Pellitorine | C_14_H_25_NO | Identified | 224.2012 | 3.92 | +H | 1 |
| 210 | 2α,3α,24-  Trihydroxyolean-11,13 (18)-dien-28-oic acid methyl ester | C_31_H_48_O_5_ | Identified | 539.3133 | 3.96 | +K | 27 |
| 211 | Piperolactam-C9:3  (2E,4E,8E) | C_20_H_23_NO_3_ | Identified | 326.1754 | 3.96 | +H | 4 |
| 212 | Armepavine | C_19_H_23_NO_3_ | Identified | 314.1744 | 3.97 | +H | 11 |
| 213 | 5-O-Methylvisamminol | C_16_H_18_O_5_ | Identified | 313.1037 | 3.97 | +Na | 2 |

**Table 2**

Total concentration of mineral and trace elements in the extracts

| S/N | Mineral elements | Concentration (mg/L) |
| --- | --- | --- |
| 1 | Sodium (Na) | 8.1 |
| 2 | Magnesium (Mg) | 19.4 |
| 3 | Potassium (Mg) | 206.2 |
| 4 | Calcium (Ca) | 2.3 |
| 5 | Chromium (Cr) | BDL (<0.5) |
| 6 | Manganese (Cr) | BDL (<0.5) |
| 7 | Iron (Fe) | BDL (<0.1) |
| 8 | Copper (Cu) | BDL (<0.5) |
| 9 | Zinc (Zn) | BDL (<0.5) |
| 10 | Arsenic (As) | BDL (<0.5) |
| 11 | Selenium (Se) | BDL (<0.5) |
| 12 | Cadmium (Cd) | BDL (<0.5) |
| 13 | Lead (Pb) | BDL (<0.5) |

BDL: Below Detection Limit
